# Aortic Geometric Alteration Associated With Acute Type B Aortic Dissection: Angulation, Tortuosity, and Arch Type

**DOI:** 10.3389/fphys.2021.708651

**Published:** 2021-08-20

**Authors:** Likun Sun, Jiehua Li, Lunchang Wang, Quanming Li, Hao He, Xin Li, Ming Li, Tun Wang, Chenglei Zhao, Xiaolong Zhang, Chang Shu

**Affiliations:** ^1^Department of Vascular Surgery, The Second Xiangya Hospital, Central South University, Changsha, China; ^2^Vascular Disease Institute, Central South University, Changsha, China; ^3^Department of Vascular Surgery, Fuwai Hospital, Chinese Academy of Medical Sciences & Peking Union Medical College, Beijing, China

**Keywords:** geometric variable, aortic angulation, aortic tortuosity, aortic arch type, acute type B aortic dissection

## Abstract

**Background:** Acute type B aortic dissection is a highly serious aortic pathology. Aortic geometric parameters may be useful variables related to the occurrence of acute type B aortic dissection (aTBAD). The aim of the study is to delineate the alteration in aortic geometric parameters and analyze the specific geometric factors associated with aTBAD.

**Methods:** The propensity score matching method was applied to control confounding factors. The aortic diameter, length, angulation, tortuosity, and type of aortic arch of the aTBAD and control group were retrospectively analyzed *via* three-dimensional computed tomography imaging created by the 3mensio software (version 10.0, Maastricht, The Netherlands). The geometric variables of true lumen and false lumen in the descending aorta were measured to estimate the severity of aortic dissection. Multivariable logistic regression models were used to investigate the significant and specific factors associated with aTBAD occurrence. The area under the receiver operating characteristic curve (AUC) was used to estimate the performance of the model.

**Results:** After propensity score matching, 168 matched pairs of patients were selected. The ascending aorta and aortic arch diameters were dilated, and the ascending aorta and total aorta lengths were elongated in aTBAD group significantly (*P* < 0.001). The ascending aorta and aortic arch angulations in the aTBAD group were sharper than those of the controls (*P* = 0.01, *P* < 0.001, respectively). The aortic arch and total aorta tortuosities were significantly higher in the aTBAD group (*P* = 0.001, *P* < 0.001, respectively). There were more type III arch patients in the aTBAD group than the controls (67.9 vs. 22.6%). The true lumen angulation was sharper than that in the false lumen (*P* < 0.01). The true lumen tortuosity was significantly lower than that in the false lumen (*P* < 0.001). The multivariable models identified that aortic arch angulation, tortuosity, and type III arch were independent and specific geometric factors associated with aTBAD occurrence. The AUC of the multivariable models 1, 2, 3 were 0.945, 0.953, and 0.96, respectively.

**Conclusions:** The sharper angulation and higher tortuosity of aortic arch and type III arch were the geometric factors associated with aTBAD in addition to the ascending aorta elongation and aortic arch dilation. The angulation and tortuosity of the true and false lumens may carry significant clinical implications for the treatment and prognosis of aTBAD.

## Introduction

Acute type B aortic dissection is an emergency and life-threatening vascular disease that involves the separation of the aortic wall layers (Nienaber and Clough, 2015). With the rapid development of interventional devices and surgical methods in the past 20 years, the overall in-hospital mortality rate of type B aortic dissection is still as high as 13% (Evangelista et al., 2018). Accordingly, it is reasonable to screen the high-risk population early for timely diagnosis and early intervention.

With the development of medical imaging and complex algorithms, the elaborate three-dimensional (3D) geometry of the aorta can be comprehended easily. Previous studies have illustrated that geometric variables such as the diameter, length, angulation, and tortuosity of ascending aorta were key risk factors for the development of type A aortic dissection (Kruger et al., 2016; Heuts et al., 2018; Gode et al., 2019; Jie et al., 2021; Saade et al., 2021). It has been gradually recognized that these geometric features may also be associated with the onset of acute type B aortic dissection (aTBAD). Currently, the published literature only focus on the relationship between the aortic diameter and length and aTBAD, while there are only few studies on whether spatial geometric factors are related to the occurrence of aTBAD (Lescan et al., 2017).

According to these observations, the aim of this study was to assess the difference in aortic geometric features between the aTBAD group and the control group, and identify specific factors associated with aTBAD occurrence.

## Methods

### Study Population

The local ethics committee approved the observational and retrospective study. Because of the retrospective and observational nature of the study, it was unnecessary to obtain written informed consent from patients. The patients diagnosed with aTBAD at the medical center between January 2018 and June 2019 were subjected to retrospective analysis. The inclusion criterion for the group with aTBAD was that the computed tomography angiography (CTA) examination was performed within 2 weeks of onset. The exclusion criteria for the group with aTBAD were as follows: history of aortic open or endovascular surgery, non-A non-B aortic dissection, isolated abdominal aortic dissection, traumatic dissection, bicuspid aortic valve, connective tissue diseases (Ehlers–Danlos syndrome, Loeys–Dietz syndrome, Marfan syndrome), bovine aortic arch, and arch branching variants (isolated left vertebral artery and aberrant right subclavian artery). The control group entailed patients who were diagnosed with non-vascular disease by CTA examination in the emergency department because of chest or back pain between January 2017 and June 2019. The exclusion criteria for the control group were history of aortic surgery, bicuspid aortic valve, connective tissue disease, and arch branching variants.

### CTA Image Processing and Analysis

All the CTA scans were acquired with a second generation dual-source computed tomography scanner (Somatom Definition Flash; Siemens Healthcare, Erlangen, Germany). CTA image datasets were collected for further analysis with the 3mensio Vascular software (version 10.0, Maastricht, Netherlands). The three-dimensional image reconstruction of the total aorta provided semi-automated vessel segmentation and lumen centerline detection, thus ensuring measurements at various spatial planes.

### Plane (P1–P7)

**Figure 1:**
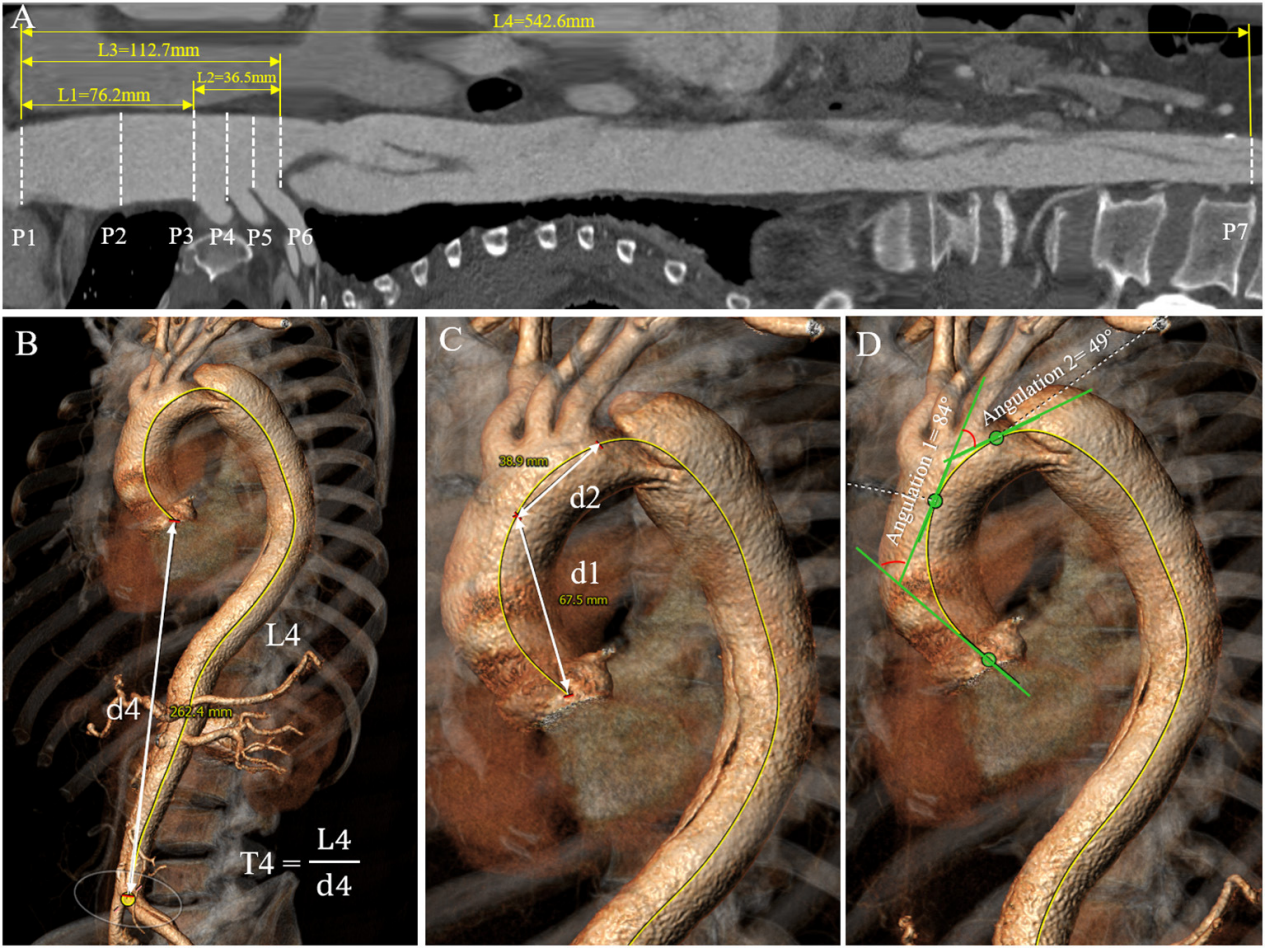
Aortic diameter, length, tortuosity, and angulation measurement process. **(A)** The length measurement of each aortic segment (L1–L4) on the stretched view and aortic diameter measurement at the landmarks D1–D6. **(B,C)** Tortuosities of the total aorta, ascending aorta and aortic arch were measured by dividing the length of the centerline (L) by the linear distance (d) between the first and last points in each aortic segment. **(D)** Angulations of the ascending aorta and aortic arch were measured as the angle formed by the tangent lines drawn along the first and last points of the aortic centerline in each aortic segment.

From the aortic root to the aortic bifurcation, a total of seven planes perpendicular to the centerline were created to measure geometric variables (Figure 1A): (P1) the sinotubular junction (STJ); (P2) the middle ascending aorta; (P3) the proximal brachiocephalic trunk (BCT); (P4) the distal BCT; (P5) the distal left common carotid artery (LCCA); (P6) the distal left subclavian artery (LSA); (P7) the aortic bifurcation.

### Diameter (D1–D6)

Aortic diameter was defined as the average of the maximum and minimum diameters measured on planes 1 to 6 (Figure 1A). The diameters of the total aortic lumen, true lumen, and false lumen in the descending aorta were calculated as the average diameters of each segment of the aorta.

### Length (L1–L4)

Aortic length was calculated from the centerline distance between the corresponding two planes above (Figure 1A). The lengths of the true lumen and false lumen in the descending aorta were obtained by establishing the centerlines in the two lumens.

### Angulation (A1–A3)

Aortic angulation was defined as the angle formed by the tangent lines drawn along the first and last points of the aortic centerline in each aortic segment by the tangent angle function in the software (Figure 1D).

### Tortuosity (T1–T4)

Aortic tortuosity was calculated by dividing the length of the centerline by the linear distance (d) between the first and last points in each aortic segment (Figures 1B,C).

### Arch Types I–III

**Figure 2:**
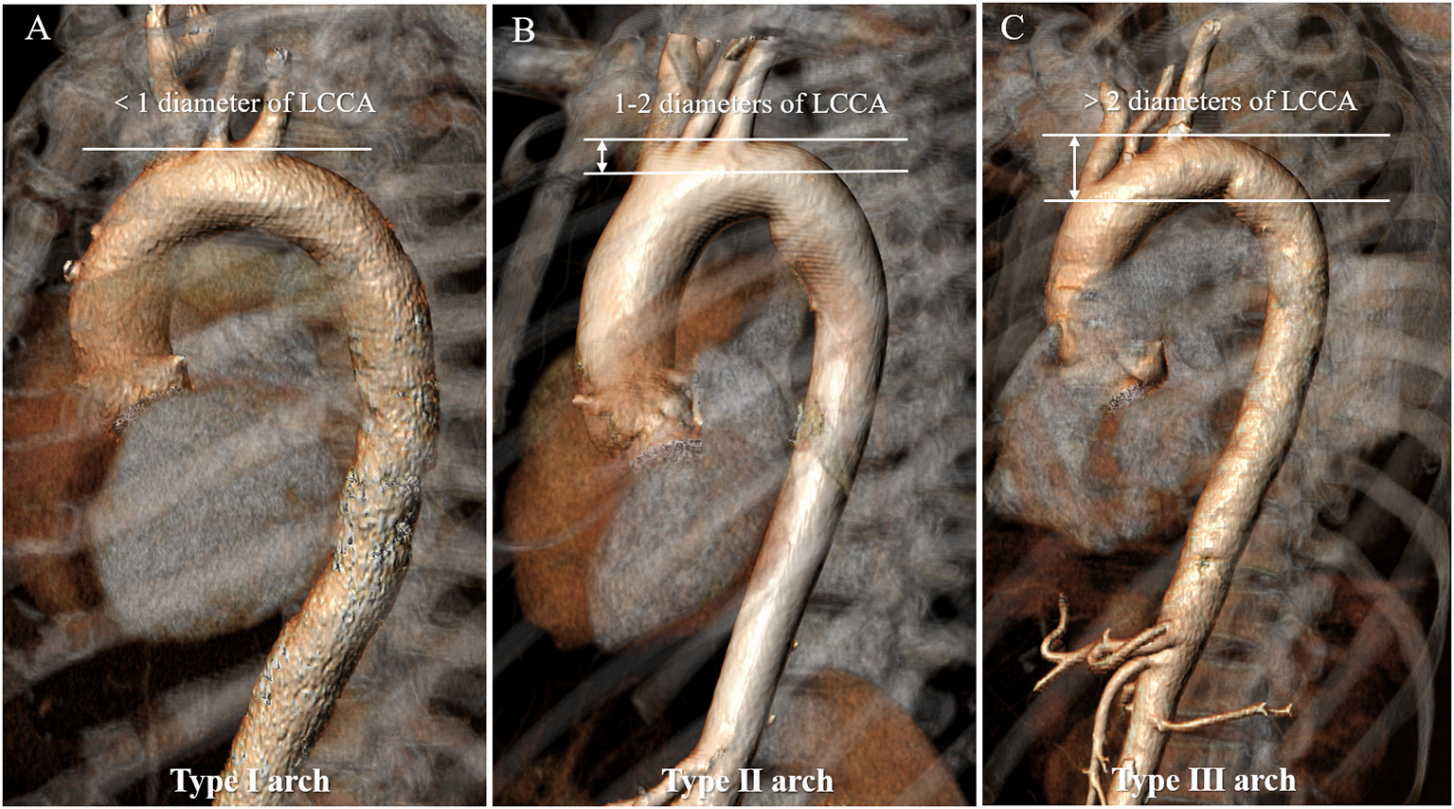
Aortic arch classification in types I to III of the normal aorta. **(A)** Type I arch: the vertical distance from the origin of the BCT to the vertex of the arch is <1 time diameter of the LCCA; **(B)** Type II arch: the distance is between 1 and 2 times the LCCA diameter; **(C)** Type III arch: the distance is more than 2 times the LCCA diameter. BCT, brachiocephalic trunk; LCCA, left common carotid artery.

Aortic arch type was divided into three types: type I arch: the vertical distance from the origin of the BCT to the vertex of the arch is <1 time diameter of the LCCA; type II arch: the distance is between 1 and 2 times the LCCA diameter; type III arch: the distance is more than 2 times the LCCA diameter (Madhwal et al., 2008) (Figure 2).

### Statistical Analysis

Categorical variables were expressed as counts and percentages, and continuous variables were reported as mean ± standard deviation (SD). We performed the Shapiro–Wilk test to assess the normality of the continuous variables and applied the Student *t*-test and Mann–Whitney test to compare the geometric variables between the aTBAD and control groups. The χ^2^-test and Fisher's exact test were performed to compare the difference in categorical variables between the two groups. Propensity score matching (PSM) was performed to control confounding factors generated by baseline differences (Benedetto et al., 2018). Patients in the control group were matched in a 1:1 ratio with the aTBAD group without replacement by nearest-neighbor matching, and the matching caliper was set at 0.05. Multivariable logistic regression models that included significant variables in the univariate analysis were used to identify geometric variables with strong and independent association with aTBAD. Statistical associations were expressed as odds ratios (ORs) with 95% confidence intervals (CIs). Receiver operating characteristic (ROC) curve and area under ROC curve (AUC) analyses were performed to quantify the performance of the model. All the data were analyzed using the *SPSS* software (version 22, IBM Inc., Armonk, NY, United States). *P* < 0.05 was considered statistically significant.

## Results

### Population Characteristics

The population characteristics of the aTBAD and control groups before PSM are shown in Table 1. A total of 523 participants were enrolled in this study, including 238 patients in the aTBAD group and 285 subjects in the control group. The male patients in the aTBAD group were more than those in the control group (80.7 vs. 59.3%, *P* < 0.001). Compared with the control group, there were more elderly patients and patients with high body mass index (BMI) and body surface area (BSA) in the aTBAD group (*P* < 0.001, *P* = 0.01 and *P* < 0.001, respectively). The proportion of hypertension in the aTBAD group was higher than that in the control group (78.6 vs. 61.8%, *P* < 0.001).

**Table 1:** Population characteristics before and after propensity score matching in study groups.

	**Before PSM**			**After PSM**		
	**aTBAD (*n =* 238)**	**Controls (*n =* 285)**	***P*-value**	**aTBAD (*n =* 168)**	**Controls (*n =* 168)**	***P*-value**
Male (%)	192 (80.7)	169 (59.3)	<0.001	143 (85.1)	137 (81.5)	0.38
Age (y)	63.7 ± 9.5	55.6 ± 10.2	<0.001	58.1 ± 9.2	56.5 ± 10.8	0.14
BMI (kg/m^2^)	28.3 ± 3.7	27.5 ± 3.6	0.01	27.1 ± 3.4	26.6 ± 3.5	0.19
BSA (m^2^)	2.1 ± 0.4	1.8 ± 0.5	<0.001	1.9 ± 0.3	1.9 ± 0.2	1
Hypertension (%)	187 (78.6)	176 (61.8)	<0.001	119 (70.8)	124 (73.8)	0.54
Hyperlipidaemia (%)	158 (66.4)	183 (64.2)	0.6	120 (71.4)	116 (69.0)	0.63
Smoking (%)	155 (65.1)	169 (59.3)	0.17	113 (67.3)	119 (70.8)	0.48

*Data are n (%) or mean ± standard deviation (SD)*.

*PSM, propensity score matching; aTBAD, acute type B aortic dissection; BMI, body mass index; BSA, body surface area*.

### Propensity Score Matching

We applied the PSM method to control confounding factors generated by the baseline differences (sex, age, BMI, BSA, hypertension, hyperlipidemia, and smoking). The PSM was performed well in balancing baseline confounders. Finally, 168 pairs were matched successfully, and there was no significant difference in population characteristics between the aTBAD group and the control group.

### Geometric Variables of Ascending Aorta and Aortic Arch

**Figure 3:**
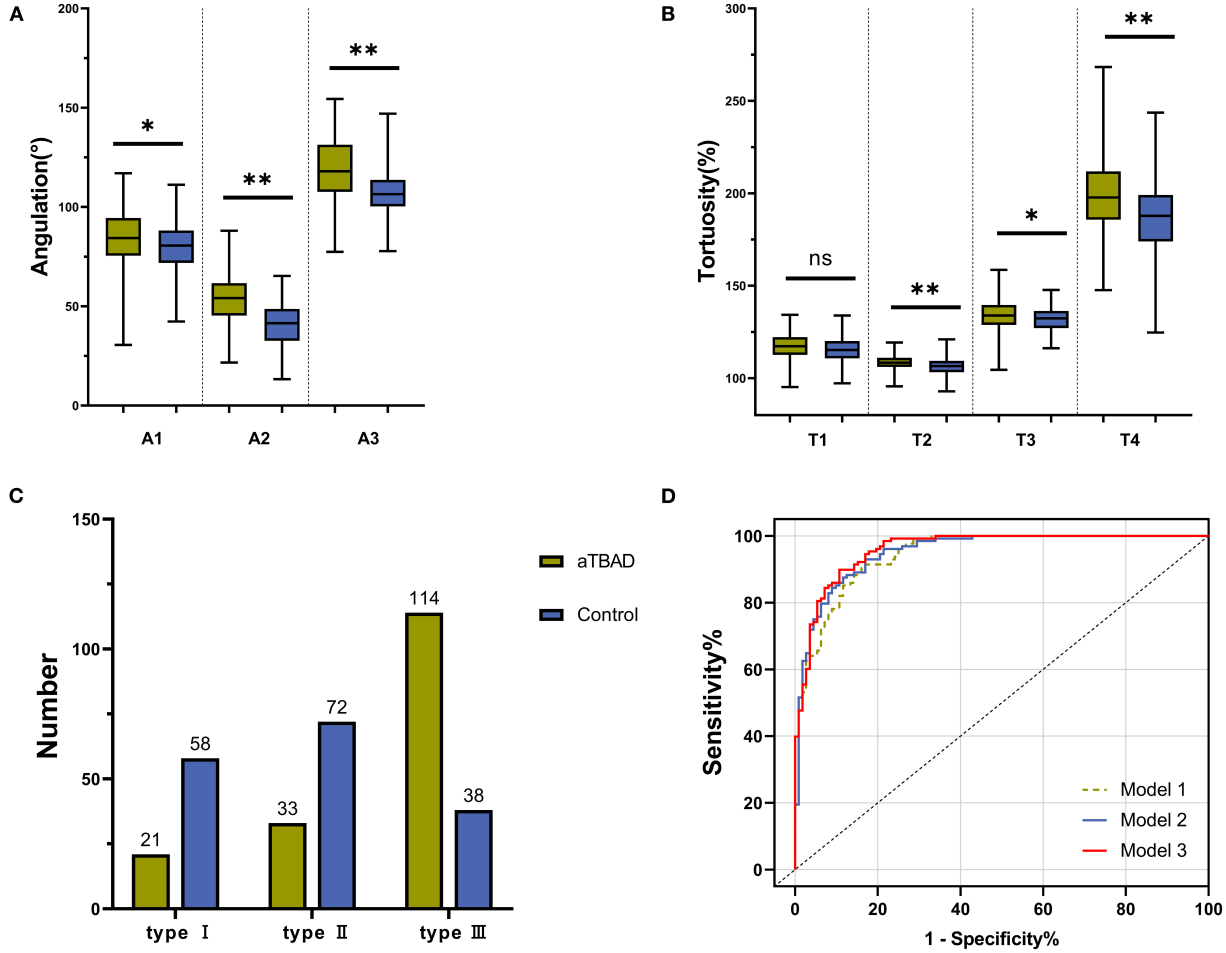
Differences in geometric variables in each aortic segment between the aTBAD and control groups. **(A,B)** Difference in aortic angulation and tortuosity in each aortic segment between the study groups. **(C)** Number of patients with aortic arch types I to III in the aTBAD and control groups. **(D)** Receiver operating characteristic (ROC) curves of the multivariable logistic regression models. *P < 0.05, **P ≤ 0.001, ns, no statistical significance.

The diameters of the sinotubular junction (STJ), mid-ascending aorta, distal ascending aorta, distal brachiocephalic trunk (BCT), distal left common carotid artery (LCCA) and distal left subclavian artery (LSA) in the aTBAD group were significantly larger than those in the control group (all *P* < 0.001, Table 2). The lengths of ascending aorta, STJ to distal LSA, and STJ to aortic bifurcation were significantly elongated in the aTBAD group (all *P* < 0.001). However, no significant difference in the length of aortic arch was found between the two groups (*P* = 0.127, Table 2). The angulations of the ascending aorta, aortic arch, and STJ to distal LSA in the aTBAD group were significantly sharper compared with those in the control group (*P* = 0.01, *P* < 0.001 and *P* < 0.001, respectively) (Table 2, Figure 3A). The tortuosity values for aortic arch, STJ to distal LSA, and STJ to aortic bifurcation in the aTBAD group were significantly higher (*P* = 0.001, *P* = 0.016 and *P* < 0.001, respectively), whereas the tortuosity of the ascending aorta showed no significant differences between the study groups (*P* = 0.066, Table 2, Figure 3B). There were more patients with type III arch in the aTBAD group (67.9%, 114/168 vs. 22.6%, 38/168), whereas the prevalence of type I and type II arches in the aTBAD group was lesser compared with the control group (χ^2^ = 69.815, *P* < 0.001, Table 2, Figure 3C).

**Table 2:** Geometric variables of the ascending aorta and aortic arch in aTBAD and control groups.

	**aTBAD** **(*n =* 168)**	**Control** **(*n =* 168)**	***t*/*χ*^2^**	***P***
**Diameter (mm)**				
STJ, D1	32.5 ± 3.9	29.2 ± 2.8	8.909	<0.001
Mid-ascending aorta, D2	35.8 ± 3.6	33.1 ± 3.4	7.067	<0.001
Distal ascending aorta, D3	34.2 ± 3.1	31.4 ± 2.7	8.828	<0.001
Distal BCT, D4	32.4 ± 3.0	29.5 ± 3.2	8.569	<0.001
Distal LCCA, D5	28.5 ± 3.4	26.3 ± 2.4	6.852	<0.001
Distal LSA, D6	27.4 ± 3.3	25.1 ± 2.6	7.096	<0.001
**Length (mm)**				
Ascending aorta, L1	74.5 ± 10.7	66.8 ± 8.2	7.403	<0.001
Aortic arch, L2	38.1 ± 7.6	37.0 ± 5.4	1.529	0.127
STJ to distal LSA, L3	114.5 ± 13.2	105.1 ± 9.7	7.438	<0.001
STJ to aortic bifurcation, L4	525.3 ± 41.6	508.3 ± 32.3	4.184	<0.001
**Angulation (°)**				
Ascending aorta, A1	84.3 ± 14.1	80.6 ± 12.1	2.581	0.010
Aortic arch, A2	53.2 ± 12.3	41.2 ± 10.9	9.464	<0.001
STJ to distal LSA, A3	118.9 ± 14.8	106.8 ± 11.3	8.423	<0.001
**Tortuosity (%)**				
Ascending aorta, T1	116.7 ± 7.2	115.3 ± 6.7	1.845	0.066
Aortic arch, T2	108.3 ± 4.2	106.5 ± 5.7	3.295	0.001
STJ to distal LSA, T3	133.9 ± 9.3	131.8 ± 6.4	2.411	0.016
STJ to aortic bifurcation, T4	198.6 ± 21.5	187.8 ± 20.3	4.734	<0.001
**Type of arch**			69.815	<0.001
Type I	21 (12.5)	58 (34.5)		
Type II	33 (19.6)	72 (42.9)		
Type III	114 (67.9)	38 (22.6)		

*Values are mean ± SD*.

*STJ, sinotubular junction; BCT, brachiocephalic trunk; LCCA, left common carotid artery; LSA, left subclavian artery; aTBAD, acute type B aortic dissection*.

### Geometric Variables of True Lumen and False Lumen in Descending Aorta

The geometric variables of the true and false lumens in the descending aorta were measured to estimate the severity of the compression of the true lumen by the false lumen (Table 3). There was no significant difference in aortic diameter and length between the true lumen and the false lumen. The angulation of the true lumen was sharper than that of the false lumen (*P* < 0.01). However, the tortuosity value of the true lumen was significantly lower than that of the false lumen (*P* < 0.001). Compared with the control group, the geometric variables of the total descending aortic lumen were significantly larger in the aTBAD group (all *P* < 0.001).

**Table 3:** Geometric variables of the descending aorta in the aTBAD and control groups.

	**aTBAD (*n* = 168)**			**Control (*n =* 168)**
	**Total aortic lumen**	**True lumen**	**False lumen**	
Descending aorta diameter (mm)	35.4 ± 3.9	18.4 ± 3.5^†^	17.8 ± 3.1	23.1 ± 2.6^‡^
Descending aorta length (mm)	438.5 ± 27.3	419.6 ± 24.5^†^	424.7 ± 26.4	406.7 ± 21.6^‡^
Descending aorta angulation (°)	106.7 ± 11.3	108.4 ± 12.1	104.5 ± 11.6^*^	99.8 ± 9.6^‡^
Descending aorta tortuosity (%)	112.4 ± 4.3	107.9 ± 4.6^†^	113.6 ± 5.1^**^	98.2 ± 3.4^‡^

† and ‡ *P < 0.001 with respect to total aortic lumen*.

* *P < 0.01*,

** *P < 0.001 with respect to true lumen*.

### Relationship Between Geometric Variables and aTBAD

Model 1 was used to investigate the geometric factors correlated with the aortic angulation. We found that the diameters of the distal ascending aorta and distal BCT, the length of the ascending aorta, and the angulation of the aortic arch were independently associated with aTBAD in model 1. Diagnostic performance, AUC, increased from 0.945 in model 1 to 0.953 in model 2 when aortic tortuosity was added as a covariate in Model 2. If the aortic arch type is also included in multivariable logistic regression model 3, diagnostic performance will continue to increase to 0.96. Model 3 identified that the angulation and tortuosity of aortic arch and type III arch were independently related to aTBAD occurrence, besides D3, D4, and L1 (Table 4, Figure 3D).

**Table 4:** Multivariable logistic regression analyses associated with the occurrence of aTBAD.

**Model**	**Variable**	**β-coefficient**	**Standard error**	**Odds ratio**	**95% confidence interval**	***P***	**AUC**
1							0.945
	D3	0.373	0.080	1.452	1.243, 1.697	<0.001	
	D4	0.320	0.072	1.377	1.195, 1.587	< 0.001	
	L1	0.132	0.027	1.141	1.081, 1.203	< 0.001	
	A2	0.097	0.022	1.101	1.056, 1.149	< 0.001	
2							0.953
	D3	0.386	0.082	1.470	1.251, 1.728	< 0.001	
	D4	0.335	0.075	1.398	1.206, 1.620	< 0.001	
	L1	0.136	0.028	1.146	1.084, 1.212	0.001	
	A2	0.096	0.022	1.101	1.055, 1.150	< 0.001	
	T2	0.109	0.045	1.115	1.022, 1.217	0.014	
3							0.960
	D3	0.370	0.087	1.448	1.222, 1.715	< 0.001	
	D4	0.339	0.081	1.403	1.197, 1.646	< 0.001	
	L1	0.122	0.030	1.130	1.066, 1.197	< 0.001	
	A2	0.105	0.024	1.110	1.060, 1.163	< 0.001	
	T2	0.132	0.049	1.142	1.036, 1.257	0.007	
	Type III	1.541	0.606	4.669	1.425, 15.300	0.011	

*aTBAD, acute type B aortic dissection; AUC, area under receiver operating characteristic curve*.

## Discussion

With computed tomography angiography imaging the diagnostic exactitude and accuracy of acute aortic dissection is excellent, ranging from 88 to 100%, and the entire dissection can be non-invasively visualized (Takahashi and Stanford, 2005). The study retrospectively analyzed computed tomography angiography (CTA) data from the study and control group to determine the independent and specific variables associated with aTBAD based on geometric characteristics. Given that patients with aortic dissection usually do not undergo a CTA examination before the onset of the disease, the CTA data of the normal aorta before dissection is difficult to obtain. In the study, we hypothesized that geometric changes in the aorta occurred prior to the onset of aTBAD, which may have a predictive value for the occurrence of the disease. It is important to note that the study findings based on the hypothesis is not yet proven. The ROC and AUC of the multivariable models should be regarded as hypothetical.

Through the analysis of the International Registry of Acute Aortic Dissection (IRAD) database, some researchers have concluded that descending aorta diameter measurement alone was inadequate for the identification of aTBAD risk, because they found that most patients with descending aorta diameter <55 mm still developed aTBAD (Trimarchi et al., 2011). Moreover, this study found that the average diameter of the total descending aortic lumen in patients with aTBAD was only 35.4 ± 3.9 mm, i.e., <55 mm, which was consistent with the above literature. Accordingly, given that the descending aorta may get deformed as dissection occurs, this study mainly employed geometric variables proximal to the region of dissection to analyze whether they are associated with aTBAD. We found that the diameters of the ascending aorta and aortic arch in the aTBAD patients were larger than those of the control group, and that the diameters of the distal ascending aorta and distal BCT were associated with aTBAD independently and specifically.

Consensus has not yet been reached in the published literature as to whether the length of each aortic segment is related to the occurrence of aTBAD. A previous study has shown that aortic arch elongation was independently associated with the development of aTBAD (Lescan et al., 2017). Another study based on a Chinese population indicated that the ascending aorta and aortic arch, specific predictors of type B aortic dissection (Cao et al., 2020), were significantly elongated compared with those of the control group. In this study, we identified that the ascending aorta was significantly longer in patients with aTBAD, and there was no significant difference in aortic arch length between the two groups. This may be because of the fact that the ascending aorta is subjected to the greater pressure of blood flow from the heart, resulting in the destruction of more elastic components (Kruger et al., 2015).

Since the diameter and length cannot adequately represent changes in the three-dimensional structure of the aorta, it is necessary to measure the spatial geometric parameters of the aorta to evaluate their correlation with aTBAD. Aortic angulation and tortuosity are the geometric parameters capable of representing the degree of vascular bending, and have not been widely studied and applied in the field of cardiovascular disease (Franken et al., 2015; Alhafez et al., 2019). Since aortic angulation and tortuosity cannot be depicted in a single plane, the 3Mensio software was used to perform automatic measurements in multiple spatial planes in this study. Shirali et al. found that the total aortic tortuosity in patients with aTBAD was higher than that in controls; however, the study did not compare the changes in tortuosity in the ascending aorta and aortic arch (Shirali et al., 2013). Cao et al. (2020) demonstrated that the tortuosity of the aortic arch and total proximal aorta in the aTBAD group were higher compared with that in controls, and that the angulations of the ascending aorta, aortic arch, and total proximal aorta were significantly larger in the aTBAD group, which were concordant with the findings of this study. The reason for the increase in aortic angulation and tortuosity may be that the upward and downward movements of the aortic arch are being restricted by the descending aorta and supra-aortic branches, respectively, and the elongation of the ascending aorta and descending aorta promote the bending of the aorta (Cao et al., 2020). Multivariable logistic regression model 1 identified that aortic arch angulation was independently associated with aTBAD. Furthermore, model 2 found that aortic arch angulation and tortuosity were both reliable geometric factors of aTBAD, and the AUC increased from 0.945 in model 1 to 0.953 in model 2.

The aortic arch classification in types I–III was originally used to predict the difficulty in cannulation of supra-aortic branches during carotid stent placement (Marrocco-Trischitta et al., 2021). Recently, some researchers have gradually recognized that aortic arch type may be a valuable parameter correlated with the onset of aTBAD. Marrocco-Trischitta et al. found that the proportion of type III arch was larger in patients who had type B aortic dissection, suggesting that type III arch configuration could be related to the high incidence of aTBAD (Marrocco-Trischitta et al., 2019). They also found that type III arch can predict endograft failure in landing zone 2 or 3 in patients undergoing thoracic endovascular aortic repair (Marrocco-Trischitta et al., 2020). In this study, the findings were similar to those of previous studies: 67.9% of patients with aTBAD had type III arch, which was significantly more than 22.6% of the control group patients. In addition, patients with aTBAD with longer aorta presented a higher proportion of type III arch, which may be due to the elongation of the ascending and descending aorta driving the apex of the aortic arch upward and distally hence forming type III arch. Besides the covariates of model 2, we incorporated the arch type as the covariate into model 3 and found that type III arch, arch angulation, and tortuosity were the significant and independent factors associated with aTBAD, and that the AUC increased to 0.96.

**Figure 4:**
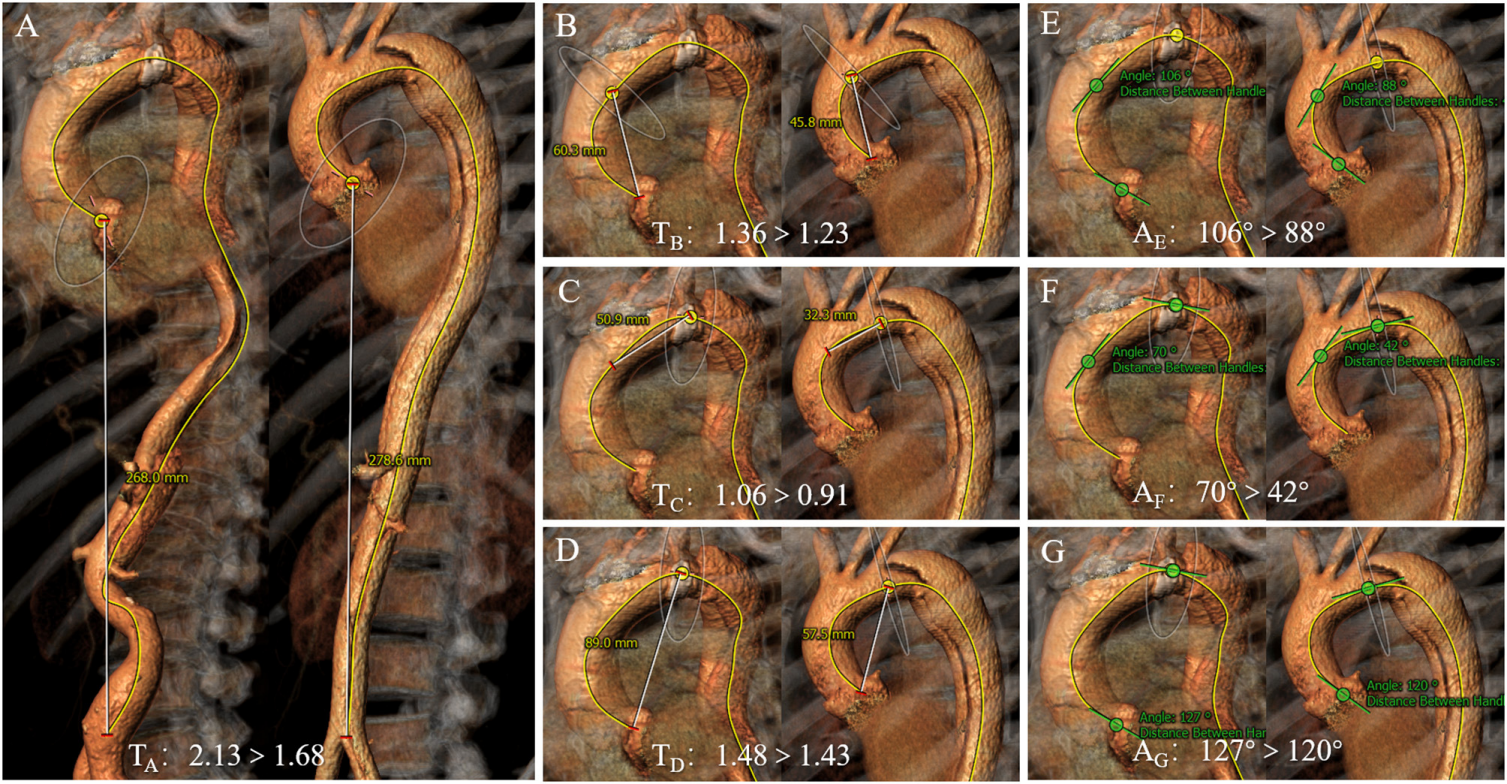
Comparison of different aortic tortuosity and angulation in patients with acute type B aortic dissection (aTBAD). **(A)** High tortuosity (left aorta) vs. low tortuosity (right aorta) of the entire aorta. **(B–D)** High tortuosity (left row) vs. low tortuosity (right row) of the ascending aorta, aortic arch, and proximal aorta. **(E–G)** High angulation (left row) vs. low angulation (right row) of the ascending aorta, aortic arch, and proximal aorta.

In order to better comprehend the pathogenesis of aortic dissection and optimize clinical treatment strategy, the geometric variables of the true lumen and false lumen of the descending aorta were measured and analyzed (Figure 4). We found that the diameter, length, angulation, and tortuosity of the total descending aortic lumen in the aTBAD group were significantly larger than those in the control group, while the angulation of the false lumen was smaller, and the tortuosity of the false lumen was higher than those of the true lumen in patients with aTBAD significantly. The difference in geometric variables between the true lumen and the false lumen may be due to the fact that the true lumen is located in the inner side and the false lumen is located in the outer side in most cases. The geometric parameters of the true and false lumens may carry significant clinical implications for the choice of thoracic endovascular aortic repair modality and stents, the occurrence of postoperative endoleak, and the long-term survival of patients.

In particular, type III arch possesses the characteristics of incremental length, angulation, and tortuosity compared with types I and II (Marrocco-Trischitta et al., 2021). Aortic elongation is actually structural remodeling caused by the longitudinal pressure exerted on the vessel wall by the blood flow (Redheuil et al., 2011; Adriaans et al., 2018). With the aggravation of aortic remodeling, the wall of the aorta becomes weak, which increases the risk of intimal tear (Akin and Nienaber, 2018; Heuts et al., 2018). Previous studies have demonstrated that the hemodynamic pressures exerted on Ishimaru zone 3 vascular wall of the aorta with type III arch were greater than those of the type I arch and type II arch (Marrocco-Trischitta et al., 2017, 2018). Moreover, increased tortuosity of the ascending aorta and aortic arch results in greater helical blood flow through the distal portion of the aortic arch and eventually increases wall shear stress (WSS) (Lescan et al., 2017). Consequently, the blood flow velocity of the aorta accelerates when the blood pressure increases suddenly. Together with the influence of the geometric factors of the aortic segments, the WSS on the vascular wall at the distal portion of the aortic arch increases rapidly, leading to a tear in the aortic intimal layer.

There are still some limitations in this study. First, the study only confirmed that the geometric variables of the aorta were related to the occurrence of aTBAD, and multicenter and prospective studies are still needed to verify whether there is a causal relationship. Second, although we adopt the PSM method to control the selection bias and potential confounders, there might be other unknown factors influencing the results. Third, in view of ethnic variation, analogous studies on other ethnicities are still needed to make comparisons and identify the degree of influence.

## Conclusions

The sharper angulation and higher tortuosity of aortic arch and type III arch configuration were the geometric factors associated with aTBAD in addition to ascending aorta elongation and aortic arch dilation. The angulation and tortuosity of the true lumen and false lumen may carry significant clinical implications for the treatment and prognosis of aTBAD. Multicenter, prospective studies are needed to determine the effect of aortic geometry on the incidence and progression of aTBAD.

## Data Availability Statement

The original contributions presented in the study are included in the article/supplementary material, further inquiries can be directed to the corresponding author/s.

## Ethics Statement

The studies involving human participants were reviewed and approved by the Medical Ethics Committee of the Second Xiangya Hospital of Central South University. Written informed consent for participation was not required for this study in accordance with the national legislation and the institutional requirements. Written informed consent was not obtained from the individual(s) for the publication of any potentially identifiable images or data included in this article.

## Author Contributions

LS and CS: conception and design. CS: administrative support. XL and QL: provision of study materials or patients. ML, HH, LW, and TW: collection and assembly of data. LS, CZ, XZ, and JL: data analysis and interpretation. All authors contributed to the article and approved the submitted version.

## Conflict of Interest

The authors declare that the research was conducted in the absence of any commercial or financial relationships that could be construed as a potential conflict of interest.

## Publisher's Note

All claims expressed in this article are solely those of the authors and do not necessarily represent those of their affiliated organizations, or those of the publisher, the editors and the reviewers. Any product that may be evaluated in this article, or claim that may be made by its manufacturer, is not guaranteed or endorsed by the publisher.
